# Optimal tuning of multi-PID controller using improved CMOCSO algorithm

**DOI:** 10.7717/peerj-cs.2453

**Published:** 2024-11-19

**Authors:** Ying Hu, Xiongyan Liu, Hao Chen

**Affiliations:** School of Computer Science and Technology, Taiyuan University of Science and Technology, Taiyuan, Wanbailin District, China

**Keywords:** Synchronous control, Constrained multi-objective optimization, Simulation, PID control, CMOCSO algorithm

## Abstract

To mitigate synchronization errors within a multi-PID controller system and enhance its resistance to interference, an improved competitive and cooperative swarm optimizer for constrained multi-objective optimization (CMOCSO) algorithm is employed to optimize the parameters of the multi-PID controller. Initially, a mathematical model representing the constrained multi-objective problem associated with the multi-PID controller is formulated. In this model, the parameters are designated as decision variables, the performance index serves as the objective function, and the stability constraints of the system are incorporated. Subsequently, an improved CMOCSO algorithm is introduced, which bifurcates the evolutionary process into two distinct stages using a central point-moving strategy; each stage employs different evolutionary techniques to accelerate convergence rates, and a novel grouping strategy is implemented to increase the learning efficiency of the population. The efficacy of the algorithm is evaluated through testing on 16 standard functions, demonstrating its effectiveness in addressing constrained multi-objective problems. Ultimately, the algorithm is applied to optimize the parameters of the multi-PID controller. The simulation results indicate that the proposed method yields superior control performance, reduced synchronization errors, and notable interference resistance capacity.

## Introduction

Advancements in control technology have led to the widespread application (Jin et al., 2024; Lopez-Sanchez & Moreno-Valenzuela, 2023; Zhang et al., 2019) of multiple proportional-integral-derivative (PID) controllers in hydraulic servo systems, particularly within sophisticated and advanced mechanical equipment. In hydraulic synchronous control systems, the interdependence of various components and the inherent complexity of the system often render a single PID controller insufficient to achieving the desired levels of precision and stability. Consequently, the implementation of multi-PID controllers for cooperative control has emerged as a viable solution to address these challenges. Nevertheless, the parameter tuning of multi-PID controllers presents a greater degree of complexity, necessitating a thorough consideration of the interactions among the controllers as well as the overall performance of the system.

Traditional methods for tuning PID controller parameters can be divided into two main categories: theoretical calculation methods and engineering tuning methods. Theoretical calculation methods rely on the system’s mathematical model to determine the PID controller parameters through theoretical analysis. For example, Zhang et al. (2016) and colleagues utilized the Rltool toolbox in MATLAB, along with the automatic control principles, to develop a PID parameter tuning approach based on root locus analysis. However, engineering tuning methods depend on practical experience and are implemented directly during control system testing, making them popular in engineering applications. One such method, known as the critical proportion method (Ziegler & Nichols, 1942), calculates PID controller parameters based on the system gain and oscillation period during critical oscillation.

Currently, a significant number of researchers are exploring the integration of multi-PID controller parameter tuning with intelligent optimization algorithms, resulting in substantial progress within the discipline. Some scholars have redefined the challenge of tuning multi-PID controller parameters as a single performance index optimization problem, utilizing single-objective optimization algorithms to ascertain optimal controller parameters. For instance, Zhang, Mao & Liu (2020) introduced a double-loop PID attitude control algorithm for balancing robots, which incorporates complementary factors alongside the Honey Badger algorithm. They developed a simulation model for the double-loop PID attitude control system applicable to balanced robots and employed the Honey Badger algorithm to optimize the parameters of multiple PID controllers, using the absolute error in integration time as the optimization criterion. Similarly, Olmez, Koca & Akpolat (2022) established a simulation model for a multi-PID controller utilizing an adaptive control approach, concentrating on integrated square error as the optimization objective, and applied the clone selection algorithm to optimize parameters across multiple PID controllers. Furthermore, Jia, Zhang & An (2022) created a simulation model consisting of two PID controllers within a dual-machine vibration synchronization system, employing a master-slave control strategy. They focused on the differences in angular velocity and phase as the optimization objective and utilized a genetic algorithm to optimize the PID parameters. Additionally, Wang et al. (2023b) proposed a design methodology for an automatic gain controller tailored for interconnected power grids utilizing flywheel energy storage, based on a cascade PI-(1+PD) structure. They implemented the particle swarm optimization (PSO) algorithm to enhance PID controller parameters, which not only simplifies the parameter tuning process but also improves the controller’s robustness against disturbances and enhances the system’s dynamic response. Moreover, Wang et al. (2023a) developed a simulation model featuring multiple PID controllers within a multi-motor synchronous control system, grounded in the framework of Relative Coupling Control. They formulated a single-objective optimization problem with performance indicators as the optimization targets and employed an artificial swarm algorithm to optimize the multi-PID parameters. However, while these studies emphasize the optimization of specific performance indices, they frequently neglect other essential performance metrics, such as steady-state error and overshoot, thereby limiting the overall control effectiveness of the system.

To address this issue, some researchers have proposed the integration of various performance indicators into a unified performance index through weighted summation, allowing for resolution within the framework of single-objective optimization to identify the most appropriate parameters for multi-PID controllers. For example, Zhang et al. (2024) developed simulation models for a crane position controller and a swing angle suppression controller in bridge cranes. They formulated a single-objective problem by combining the time absolute error of both PID controllers, applying weights to the control values to mitigate excessive control margins, and incorporating an overshoot penalty coefficient. An improved sparrow search algorithm was employed to optimize PID controller gains, minimizing negative impacts on payload oscillation and trolley positioning during bridge crane operations. Similarly, Feng et al. (2024) introduced an improved PSO algorithm to address parameter optimization of multiple PID controllers in various hydraulic systems. They utilized the recursive least squares identification method to derive the actual mechanism model and employed the weighted summation of four performance indicators to optimize the PID controller parameters, thereby enhancing the trajectory tracking accuracy of the system. However, this approach presents a significant limitation: the selection of weights can substantially influence the final optimization outcomes. Variations in weight assignments yield divergent optimization results, necessitating careful consideration in the selection of weights to ensure that optimal parameters adequately fulfill the multiple performance criteria of the system.

Numerous scholars have approached the challenge of parameter tuning in multi-PID controllers by framing it as a multi-objective optimization problem, utilizing various multi-objective optimization algorithms to consider a wider array of system performance metrics. For instance, Zhang, Mao & Liu (2020) applied the NSGA-II multi-objective optimization algorithm to refine the parameters of the PID-I controller, specifically addressing the delay issues inherent in photoelectric tracking systems. This algorithm emphasized closed-loop bandwidth and step response performance as key optimization objectives, employing a non-inferior ranking strategy to simultaneously enhance the open-loop gain and bandwidth of the system, thereby improving the controller parameters. In a similar vein, Yin et al. (2022) created a simulation model for a multi-PID controller within a steam compression refrigeration unit, defining the multi-objective fitness function as the minimization of absolute integral time error, adjustment time, and steady-state error. They utilized a modified multi-objective artificial fish swarm algorithm to optimize six parameters across two PID controllers, resulting in a significant improvement in system control quality. Furthermore, Milašinović et al. (2022) developed a simulation model of the multi-PID controller based on the open channel hydraulic model of control theory, targeting root-mean-square error, process error, assimilation time ratio, and total correction volume as multi-objective optimization criteria. The non-dominated sorting genetic algorithm II (NSGA-II) was employed to fine-tune the parameters of the two-stage PID controller. To maintain high control quality in the regulation systems of pumped storage units, Jiang, Xiu & Yu (2024) proposed a fractional order PID controller, which offers greater applicability and flexibility compared to conventional PID controllers, utilizing the integral of time-weighted absolute error index under low and medium-high water head conditions as the optimization objective. An advanced multi-objective particle swarm optimization (PSO) approach was introduced for the tuning of PID controller parameters. Additionally, Hu, Lv & Yuan (2024) constructed a multi-PID controller simulation model based on a master-slave synchronous control framework, concentrating on optimization objectives such as master-slave synchronization error, adjustment time, overshoot, and integral square error. They employed an improved multi-objective evolutionary algorithm based on decomposition to optimize the parameters of the master-slave PID controller. Moreover, Wang et al. (2024) developed a multi-objective optimization strategy for the fractional order control of electromagnetic suspension systems, constructing a multi-body dynamics model for maglev vehicles that incorporated fractional order PID control. The researchers selected the Sperling index and variations in vertical suspension clearance as optimization criteria and utilized multi-objective optimization techniques to compute the Pareto frontier, thereby identifying the optimal set of control parameters. Lastly, Yang et al. (2024) introduced an optimization strategy that integrates the spiral model of the whale optimization algorithm with the multi-objective marine predator algorithm, aiming to minimize the integral time square error, the integral time absolute error, and the rate of change of deviation to optimize the PID parameters. The optimized PID controller developed exhibited a more rapid frequency response and enhanced resilience to load disturbances within the power system, thereby significantly improving the overall stability of the system. A review of the existing literature reveals that, although numerous studies have established multi-objective optimization models to comprehensively evaluate various performance metrics of the system, these models are not without their limitations. A primary concern is that they often prioritize time-domain indices during the design phase, while giving insufficient attention to frequency-domain performance indices. This oversight may result in an incomplete assessment of control quality. Therefore, there is a potential to refine the multi-objective optimization model by incorporating both frequency-domain and time-domain methodologies, which would enhance the comprehensiveness and effectiveness of system control.

This study formulates the parameter tuning challenge of the multi-PID controller as a constrained multi-objective optimization problem (CMOP) characterized by a seven-dimensional target space, a twelve-dimensional decision space, and three constraints. There are two types of constrained multi-objective evolutionary algorithms (CMOEA) for solving CMOP: the first one is based on multi-population strategy; the second algorithm is based on multi-stage strategy. The weak coevolution framework introduced by Tian et al. (2020) enhances the convergence and diversity of the primary population in the target space by incorporating primary populations to support the main population. However, the efficacy of the coevolution framework is significantly influenced by the spatial relationship between the unconstrained pareto front (PF) and the constrained pareto front (CPF). When these two fronts are distanced from each other, the primary populations’ contribution is diminished, and the foundational optimization algorithm, NSGA-II, proves inadequate for large-scale problems. Ma et al. (2021) proposed a multi-stage CMOEA algorithm that addresses CMOP with intricate feasible regions by incrementally introducing constraints and managing them separately across various evolutionary stages. Nevertheless, the multi-stage CMOEA approach may present complexities in constraint ordering and composition, necessitating careful timing of phase transitions to prevent the algorithm from succumbing to local optima or stagnation. In contrast, Ming et al. (2022a) introduced a competitive and cooperative group optimization algorithm (CMOCSO), which uses constraint relaxation techniques to explore the CPF through a competitive group optimizer while disregarding the constrained PF through a cooperative group optimizer. This method effectively balances convergence, diversity, and feasibility, making it particularly well suited for complex constrained optimization challenges. The CMOCSO algorithm not only accommodates multiple objectives and constraints simultaneously but also utilizes the basic optimization algorithm, the competitive swarm optimizer, which demonstrates superior convergence speed and stability compared to the previously mentioned algorithms. Furthermore, the CMOCSO algorithm has been successfully implemented in various domains, including the multi-objective deterministic optimal reactive power dispatch problem (Bhurt et al., 2023) and practical continuous mechanical design problems (Ming et al., 2024).

This research presents an improved CMOCSO (ICMOCSO) algorithm, aimed at improving its efficacy in traversing intricate PF while simultaneously decreasing execution time in real-world applications. The ICMOCSO algorithm is utilized to tackle the parameter tuning challenge associated with multi-PID controllers. The key contributions of this study are summarized as follows:

1. The parameter tuning issue of the multi-PID controller system is transformed into a constrained multi-objective problem. The controller parameters are set as decision variables, and the optimization objectives encompass seven key performance indicators. Meanwhile, the amplitude margin, phase margin, and overshoot are regarded as constraints.

2. The ICMOCSO algorithm is proposed, employing the center-point moving strategy to divide the optimization process into two stages. In the first stage, both the main population and the cooperative population evolve concurrently. In the second stage, only the main population is utilized to search for the optimal solution set.

3. During the evolution process of the main population, a grouping strategy is introduced to enhance the global search capability and the local fine-tuning ability of the algorithm. The validity of ICMOCSO in constrained seven-objective problems is verified by comparing it with 16 seven-objective ZXH_CF standard functions.

4. The improved algorithm is applied to the parameter tuning problem of multi-PID controllers and is compared with other methods. The results indicate that the ICMOCSO method achieves a more superior control effect, reduces the synchronization error among systems, and exhibits excellent anti-interference ability.

The subsequent sections of this article are structured as follows: ‘Multi-PID Controller Model’ delineates the control strategy employed by multiple PID controllers within the hydraulic synchronous control system, along with the specific parameters associated with the executing components. ‘Constrained Multi-Objective Problem’ presents the mathematical framework for the multi-PID controller, encompassing optimization objectives, constraints, individual coding, and the range of parameter searches. ‘Proposed ICMOCSO Approaches’ provides a detailed explanation of the ICMOCSO change response mechanism and demonstrates the algorithm’s efficacy. ‘Numerical Simulation’ addresses the parameter settings for the simulation experiments aimed at tuning the multi-PID controller, as well as the analysis of results and comparisons with other methods. Finally, ‘Conclusions’ summarizes the findings of the article based on the numerical simulations conducted.

## Multi-pid controller model

In a hydraulic synchronous control system, the collaboration of multiple PID controllers is essential to ensure stable and efficient system performance. Common strategies for synchronous control include equal-state, master-slave, and cross-coupling approaches. The equal-state synchro-driven method tends to yield high error accuracy due to the parallel configuration of subsystems. However, when the hydraulic system incorporates more than two hydraulic branches, the cross-coupling control strategy can introduce complexities that may adversely affect the performance of the hydraulic synchronous control system. The master-slave control approach utilizes the output from the master structure as the input for the slave structure. Although this method may result in a certain degree of lag during the tracking process, such lag can be effectively mitigated through the implementation of PID control strategies or other advanced control technologies (Guan, 2023). Given these considerations, the master-slave control mode has been selected for the simulation model of the hydraulic synchronous control system. This decision is informed by a thorough evaluation of system performance and complexity, with the objective of achieving efficient and stable hydraulic synchronous control.

**Figure 1:**
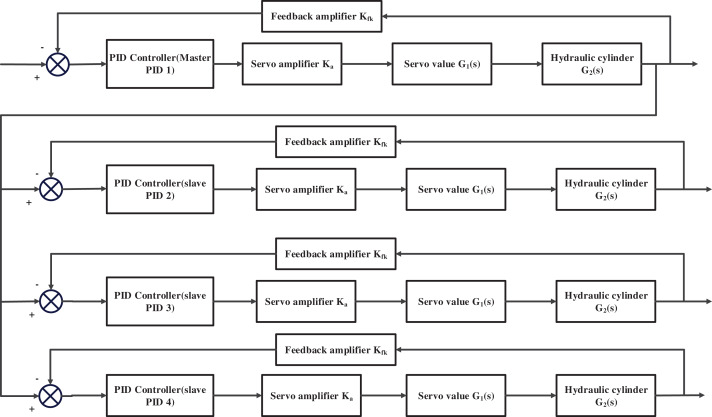
Electro-hydraulic servo synchronous control system.

The electro-hydraulic servo system consists mainly of core components such as PID controllers, amplifiers, feedback amplifiers, servo valves, and hydraulic cylinders. Based on the mathematical model of each component, we constructed the control flow chart of a multi-PID controller in an electro-hydraulic servo synchronous system, as shown in Fig. 1.

Before constructing the model, we refer to the relevant literature Guo et al. (2022) to obtain the precise parameters of each actuator element, as shown in Table 1. The transfer function model ${G_1}(s)$ (Eq. (1)) for the servo valve and ${G_2}(s)$ (Eq. (2)) for the hydraulic cylinder.

**Table 1:** Hydraulic cylinder and servo valve parameters.

Number	Parameters	Numerical value
1	Amplification factor of the servo amplifier ( $K_a$)	0.004
2	Amplification factor of the feedback amplifier ( ${K_{fk}}$)	90.9
3	Servo valve flow gain ( ${K_{f}}$)	0.021 m/A
4	Natural frequency of the servo valve ( ${W_f}$)	753.6 rad/s
5	Damping ratio of the servo valve ( ${\varsigma_f}$)	0.7
6	Effective piston area ( ${A _p}$)	0.00489 ${A_p}$
7	Hydraulic natural frequency ( ${W_h}$)	314 rad/s
8	Hydraulic damping ratio ( ${\xi_h}$)	0.2

(1) $${G_1}(s) = {{{k_{\mathrm{f}}}} \over {{{{s^2}} \over {w_f^2}} + {{2{\varsigma _f}} \over {{w_f}}}s + 1}},$$

where $s$ denotes the laplace operator, ${K_f}$ denotes the flow gain of the servo valve, ${W_f}$ denotes the natural frequency of the servo valve, and ${\varsigma _f}$ denotes the damping ratio of the servo valve.

(2) $${G_2}(s) = {{1/{A_p}} \over {s({{{s^2}} \over {w_h^2}} + {{2{\xi _h}} \over {{w_h}}}s + 1)}},$$

where $s$ denotes the laplace operator, ${A_p}$ denotes the effective piston area, ${W_h}$ denotes the hydraulic natural frequency, and ${\xi _h}$ denotes the hydraulic damping ratio.

**Figure 2:**
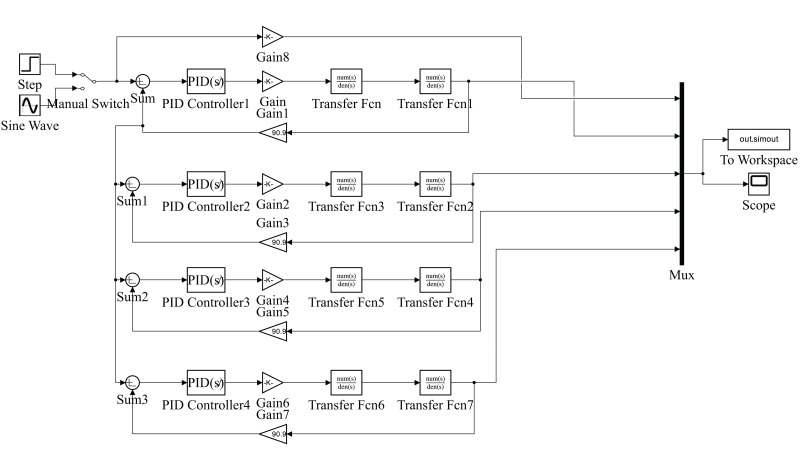
Simulation model of multi-PID controller for electro-hydraulic servo synchronous control system.

Using the Simulink toolbox in MATLAB, we established the simulation model of the multi-PID controller for the electro-hydraulic servo synchronous control system (as shown in Fig. 2).

## Constrained multi-objective problem

The objective of optimizing the parameters of a multi-PID controller is to concurrently improve the dynamic performance, stability and robustness of the system through the adjustment of PID controller parameters. This study emphasizes seven critical performance indicators of the system as optimization objectives while also incorporating constraints to ensure the robustness and stability of the system. These elements are integrated into a constrained multi-objective optimization problem aimed at optimizing the parameters of a multi-PID controller for an electro-hydraulic servo-synchronous control system.

### Optimization objectives

PID controllers, which are designed to minimize time-domain performance metrics, have been extensively employed in control engineering. Historically, some researchers have concentrated on optimizing PID parameters with a singular objective; however, this methodology is inadequate for identifying the optimal PID parameters. Therefore, a holistic assessment of multiple factors is essential. This article considers several standard performance metrics simultaneously to evaluate the efficacy of electro hydraulic servo synchronous control systems.

The system overshoot quantifies the percentage by which the peak value of the step response surpasses its final steady-state value. It is computed as illustrated in Eq. (3).

(3) $${{\mathrm{J}}_1} = \sigma = \sum\limits_{i = 1}^n {{{c_i^{\max } - {c_i}(\infty )} \over {{c_i}(\infty )}}} ,$$

where $c$ represents the step response curve of the system, ${c_{max}}$ denotes the maximum value of the step response, $c(\infty )$ denotes the final steady state value of the step response, ${c_i}$ denotes the step response curve associated with the i-th PID controller within the system, and $n$ signifies the number of PID controller subsystems in the system.

The adjustment time is defined as the minimum duration required for the step response to attain and remain consistently within a 2% error margin of the final steady-state value. The calculation is shown in Eq. (4).

(4) $${{\mathrm{J}}_2} = {t_s} = \sum\limits_{i = 1}^n {\left\{ {\left. t \right|\left| {{{{c_i}(t) - {c_i}(\infty )} \over {{c_i}(\infty )}}} \right|{ \lt}\,0.02} \right\}} ,$$

where $c$ represents the step response curve of the system, $c(\infty )$ denotes the final steady state value of the step response, ${c_i}$ denotes the step response curve associated with the i-th PID controller within the system, $t$ denotes the system’s simulation time, and $n$ stands for the number of PID controller subsystems.

The rise time is defined as the duration necessary for the step response to first reach the final steady-state value. It is calculated as shown in Eq. (5).

(5) $${{\mathrm{J}}_3} = {t_r} = \sum\limits_{i = 1}^n {\left\{ {\left. t \right|{c_i}(t) - {c_i}(\infty ) = 0} \right\}} ,$$

where $c(\infty )$ denotes the final steady state value of the step response, ${c_i}$ denotes the step response curve associated with the i-th PID controller within the system, $t$ denotes the simulation time of the system, and $n$ denotes the number of PID controller subsystems in the system.

The peak time is defined as the time required for the step response to surpass the final steady-state value for the first time. It is calculated as shown in Eq. (6).

(6) $${{\mathrm{J}}_4} = {t_m} = \sum\limits_{i = 1}^n {\left\{ {\left. t \right|{c_i}(t) > {c_i}(\infty )} \right\}} ,$$

where $c(\infty )$ denotes the final steady state value of the step response, ${c_i}$ ddenotes the step response curve associated with the i-th PID controller within the system, $t$ denotes the simulation time of the system, and $n$ denotes the number of PID controller subsystems in the system.

The steady-state error quantifies the difference between the theoretical and actual step response values, computed as outlined in Eq. (7).

(7) $${{\mathrm{J}}_5} = se = \sum\limits_{i = 1}^n {\left| {out - {c_i}(\infty )} \right|} ,$$

where $c(\infty )$ denotes the final steady state value of the step response, ${c_i}$ denotes the step response curve associated with the i-th PID controller within the system, $out$ is the ideal output value of the system, $t$ denotes the simulation time of the system, and $n$ denotes the number of PID controller subsystems in the system.

The synchronization error assesses the deviation between the master and slave structures in systems utilizing master-slave synchronization control, calculated according to Eq. (8).

(8) $${{\mathrm{J}}_6} = {e_{ss}} = \sum\limits_{i = 1}^{n - 1} {\int\limits_{}^{} {{c^m}(\infty ) - c_i^s(\infty )} } dt,$$

where $c(\infty )$ denotes the final steady state value of the step response, ${c_i}$ denotes the step response curve associated with the i-th PID controller within the system, $t$ denotes the simulation time of the system, $n$ denotes the number of PID controller subsystems in the system, ${c^m}$ denotes the step response curve of the PID controller of the master structure, and ${c^s}$ denotes the step response curves of the PID controllers of the slave structure.

The integral time squared error not only mitigates excessively large signal values that may exceed the amplitude of the PID controller, but also avoids the error rate that may cause delays in the sensors in the controller (Lin et al., 2022). The calculation is illustrated in Eq. (9).

(9) $${{\mathrm{J}}_7}{\mathrm{ = ITSE = }}\sum\limits_{{\mathrm{i = 1}}}^{\mathrm{n}} {\int_0^\infty {te_i^2(t)} dt} ,$$

where $e(t)$ denotes the error between the ideal and actual output values of the system at time $t$, $i$ denotes the i-th PID controller subsystem within the system, $t$ signifies the simulation time, and $n$ denotes the number of PID controller subsystems in the system.

### Constraints set

In order to define parameters for system robustness, two essential factors are taken into account: amplitude margin and phase margin. The phase margin (*PM*) refers to the permissible additional phase lag at the gain crossover frequency that is necessary to achieve critical stability within the system. Conversely, the gain margin (*GM*) represents the maximum allowable increase in the system’s gain while still preserving critical stability. As outlined in Hu (2013), the *PM* is generally observed to fall within the range of 30 to 60 degrees, whereas *GM* is typically expected to exceed 6 dB.

The magnitude of system overshoot is a critical consideration in the assessment of system stability. An excessive overshoot may result in instability and adversely affect the system’s normal functioning. Consequently, it is imperative to establish a constraint on the level of overshoot. By regulating overshoot, one can enhance system stability during operation and mitigate unwarranted fluctuations and atypical behaviors.

The system overshoot constraint calculation is calculated by Eq. (10).

(10) $${\sigma ^{\mathrm{c}}} = \mathop {\max }\limits_{i = 1..n} \left({{c_i^{\max } - {c_i}(\infty )} \over {{c_i}(\infty )}}\right),$$

where $c$ denotes the step response curve of the system, ${c^{max}}$ denotes the maximum value of the step response, $c(\infty )$ denotes the final steady-state value of the step response, ${c_i}$ denotes the step response curve associated with the i-th PID controller within the system, and $n$ denotes the number of PID controller subsystems in the system.

### Individual coding and parameter search range

The ‘Multi-PID controller model’ discussed in the preceding section illustrates that the simulation framework comprises four PID controllers, each characterized by three parameters: proportional, integral, and derivative. Consequently, the decision space for the constrained multi-objective optimization is represented in 12 dimensions. Specifically, the decision variables are organized as [ ${K_{p1}}$, ${K_{i1}}$, ${K_{d1}}$, ${K_{p2}}$, ${K_{i2}}$, ${K_{d2}}$, ${K_{p3}}$, ${K_{i3}}$, ${K_{d3}}$, ${K_{p4}}$, ${K_{i4}}$, ${K_{d4}}$], where ${K_{pj}}$, ${K_{ij}}$, and ${K_{dj}}$ denote the proportional, integral, and derivative parameters of the j-th PID controller, respectively. The decision space is encoded using real numbers, with each PID parameter expressed as a real number. The search space is established based on parameters referenced in the literature (Zhang et al., 2016) and is defined by extending the range both upwards and downwards by a specified multiple (for instance, from 0.5 to 1.5 times the original value). For instance, if the PID parameter value calculated from the literature is denoted as *X*, the search range can be delineated as $[0.5X,1.5X]$. Should it be observed that the majority of the optimized PID parameter results tend to cluster near the boundaries of the search range, the multiplicative factor of the search range may be further modified to broaden the search space.

In summary, the constrained multi-objective optimization model proposed in this article is calculated by Eq. (11).

(11) $$\matrix{ {\min\; f(x) = ({{\mathrm{J}}_1},{{\mathrm{J}}_{\mathrm{2}}},{{\mathrm{J}}_{\mathrm{3}}},{{\mathrm{J}}_{\mathrm{4}}},{{\mathrm{J}}_{\mathrm{5}}},{{\mathrm{J}}_{\mathrm{6}}},{{\mathrm{J}}_{\mathrm{7}}}),} \hfill \cr {x = [{K_{p1}},{K_{i1}},{K_{d1}},{K_{p2}},{K_{i2}},{K_{d2}},{K_{p3}},{K_{i3}},{K_{d3}},{K_{p4}},{K_{i4}},{K_{d4}}],} \hfill \cr {s.t.\quad GM > {g_{\min }},{{\mathrm{p}}_{\max }} > { {PM}} > {{ {p}}_{\min }},{\sigma _{\max }} > {\sigma ^{\mathrm{c}}},} \hfill \cr }$$

where ${J_1}$ is the overshoot, ${\sigma ^c}$ is the overshoot constraint, ${\sigma _{max}}$ is the upper bound of the overshoot, ${J_2}$ is the rise time, ${J_3}$ is the regulation time, ${J_4}$ is the peak time, ${J_5}$ is the steady state error, ${J_6}$ is the error between the two subsystems, ${J_7}$ is the integral squared error, *GM* and *PM* denote the gain margin and phase margin of the system, respectively, and ${g_{min}}$, ${p_{max}}$, ${p_{min}}$ denote the upper and lower bounds of the respective constraints.

## Proposed icmocso approaches

### Two-stage policy

**Figure 3:**
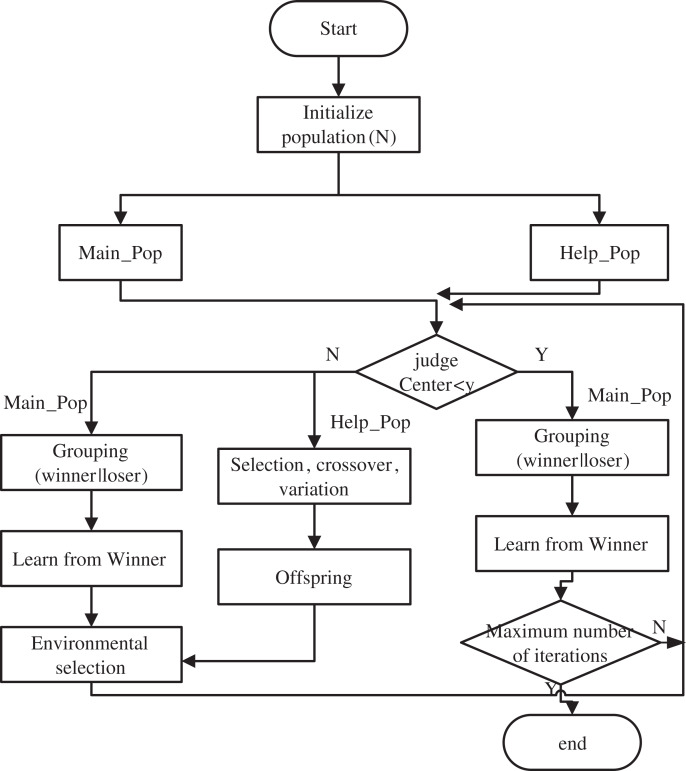
General principle of improved CMOCSO.

ICMOCSO is an improved CMOCSO algorithm that utilizes a two-stage strategy. In the traditional CMOCSO, two populations coexist throughout the evolutionary process, resulting in increased algorithm runtime. However, ICMOCSO divides the entire evolutionary process into two stages by introducing a centroid movement strategy. In the initial phase, a new grouping strategy and environment selection method are implemented, accelerating the convergence of the main_Pop. Simultaneously, coevolution is achieved by sharing the offspring of main_pop and help_pop. In the second phase, emphasis on the evolution of the main_Pop further accelerates the entire algorithm. The primary principle of the ICMOCSO algorithm is illustrated in Fig. 3.

### Combined two-stage strategy

In traditional CMOCSO, the presence of two continuously evolving populations throughout the evolutionary process often results in prolonged runtime and increased computational costs when addressing real-world problems. Conversely, ICMOCSO algorithm is structured into two distinct phases. The initial phase introduces a variety of novel strategies, including grouping strategies and environmental selection methods, aimed at enhancing the convergence of the main_Pop while facilitating coevolution with the help_Pop. During this phase, the main_Pop employs $\varepsilon$-constraint techniques (Song et al., 2024) to conduct a global search for the CPF, while the help_Pop focuses on identifying the PF within the objective space. This strategic design mitigates the risk of the main_Pop prematurely converging to local optima, thereby promoting the global convergence of the algorithm. In the environmental selection process, the main_Pop selects optimal solutions from the offspring generated by both the main_Pop and help_Pop, ensuring the continued evolution of the main_Pop. Once the centroid of the main_Pop reaches a predetermined distance, the algorithm transitions into the second phase.

In the second phase, since the first phase has already determined the approximate range of the CPF, only the main_Pop continues to evolve under all constraints. The focus of this phase is to accelerate convergence and reduce the algorithm’s cost. By focusing on the evolution of the main_Pop, the algorithm can find the optimal solutions faster, effectively shortening the runtime while ensuring search accuracy and effectiveness.

The $\epsilon$-constraint technique is an approach utilized to manage constraints dynamically by incorporating a relaxation factor, $\epsilon$, within the optimization process. This methodology permits the initial relaxation of stringent constraint requirements during the early phases of the optimization search, thereby broadening the search space and augmenting the diversity of potential solutions. As the optimization process advances, the value of $\epsilon$ is systematically reduced, which progressively strengthens compliance with the constraints. This mechanism effectively directs the algorithm towards convergence within the feasible solution region that fully adheres to the established constraints. As outlined in reference Fan et al. (2019), the updating process for $\epsilon$ is as follows:

(12) $$\matrix{ {\epsilon (t) = \left\{ {\matrix{ {{\phi ^t}(x),}\hfill & {if} & {t = 0,} \hfill\cr {(1 - \tau )\epsilon (t - 1),}\hfill & {if} & {{r_t}\;{\lt}\;\alpha ,}\hfill \cr {{\phi _{\max }}{{(1 - {t \over {{T_c}}})}^{cp}},}\hfill & {if} & {{r_t}, \ge \alpha ,}\hfill \cr {0,}\hfill & {if} & {t \ge {T_c},}\hfill \cr } } \right.} \hfill \cr }$$

where ${\phi ^t}(x)$ is the total constraint violation of the top individuals in the population, ${r_t}$ is the feasible ratio of population, $\alpha$ and $\tau$ are two control parameters, ${\phi _{\max }}$ is the maximal constraint violation of population found so far, and ${T_c}$ controls the maximal generation that $\epsilon$ works, while $cp$ reducing the $\epsilon$ value in the case of ${r_t} \ge \alpha$.

### Center-point movement strategy

Currently, many two-phase algorithms use a semi-partitioning method to distinguish stages, considering the first $N\%$ of the entire search process as one phase and the remaining part as another phase. However, the ICMOCSO algorithm differs from this common semi-partitioning method in that it features an automatic phase transition mechanism. This mechanism determines the current phase by monitoring the movement distance of the central point of the main population. When the movement distance of the main population’s central point is sufficiently small, it indicates that the population has found a relatively stable optimal solution in the solution space and the evolutionary process has reached a saturation state. At this point, continuing the search in the current phase may not yield further improvements, so the algorithm can automatically transition to the next phase to better explore other parts of the solution space. The central point of the population aggregates and summarizes the information of all individuals in the population. By analyzing the position and movement of the central point, one can obtain global information about the population in the solution space. The calculation formula for the central point is calculated by Eq. (13).

(13) $${C_t} = {1 \over {\left| {MainPop} \right|}}\sum\limits_{x \in MainPop} {F(x),}$$

where *C* represents the central point of the main population, $t$ represents the number of iterations of the algorithm, $|MainPop|$ represents the number of individuals in the main population, $x$ represents an individual in the main population, and $F(x)$ represents the fitness value of each individual.

If the Euclidean distance between the central points of the main population in two iterations exceeds a critical value, the search process will remain in the first phase; otherwise, it will automatically transition to the next phase. The formula for the central point movement strategy is calculated by Eq. (13).

(14) $$\left\{ {\matrix{ {{\mathrm{stage1}}:\left| {|{{\mathrm{C}}_t} - {C_{t - 1}}} \right|| > \theta ,}\hfill \cr  {{\mathrm{stage2}}:\left| {|{{\mathrm{C}}_t} - {C_{t - 1}}} \right|| \le \theta ,} \cr  } } \right.$$

where *C* represents the central point of the main population, $t$ represents the number of iterations of the algorithm, $\theta$ represents the minimum value of the central point movement, $stage1$ and $stage2$ represent the two phases of the improved CMOCSO, and || || calculates the Euclidean distance between the two central points.

### Grouping strategy

**Figure 4:**
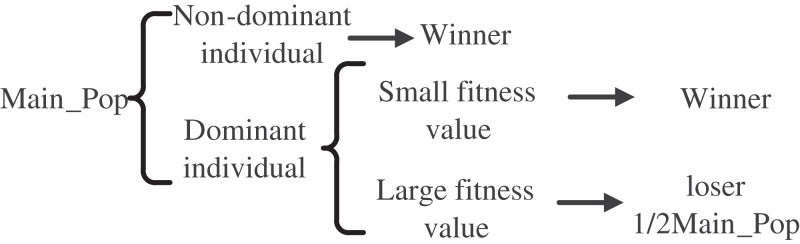
General principle of improved CMOCSO.

The ICMOCSO algorithm presents an innovative grouping strategy that integrates pareto dominance with fitness value comparison. In contrast to the CMOCSO approach, where individuals are randomly grouped and subsequently paired for mutual learning based on their fitness values, the ICMOCSO method categorizes particles into winning and losing groups through the application of pareto dominance and fitness value assessments. In this framework, non-dominated individuals are immediately classified as winners. Among the remaining individuals, those exhibiting lower fitness values are selected to join the winning group, thereby maintaining an equal distribution of individuals across both groups. Following the completion of this comparison, the winners advance directly to the subsequent generation, while the losers engage in a learning process from the winners. This strategic approach not only improves the search efficiency of the algorithm but also enhances the overall quality of the solutions produced. The individual comparison process is shown in Fig. 4.

The steps of the grouping strategy in this algorithm are as follows: Firstly, individuals in the population are divided into different front groups through non-dominated sorting. Then, individuals are classified into winner or loser groups based on whether they are in the first layer of the pareto front. If no individuals are in the first layer of the pareto front, individuals are allocated to the two groups based on fitness value comparison. If the number of individuals on the pareto front exceeds half of the total population, the top $N/2$ individuals with the smallest fitness values are selected as the winner group, and the remaining individuals are the loser group. If the number of individuals on the pareto front is less than half of the total population, the top $N/2$ individuals with the highest fitness values are selected as the loser group, and the remaining individuals are the winner group.

Each individual is allocated a fitness value, which is determined by two primary factors: the amount of the solution dominated by other solution $\epsilon$ constraints, and the crowding distance that measures the proximity of the solution to other solutions. The precise methodology for this calculation is presented in Eq. (15).

(15) $$F(i) = |\{ j \in Q|j{ \prec _\epsilon }i\} | + d, $$

where || denote the cardinality of a set, $d$ signify the crowding distance between two solutions, *Q* represent the current solution set, and the notation $j{ \prec _\epsilon }i$ indicate that solution $j$ $\epsilon$-constraint dominates solution $i$. For two solutions, $x$ and $y$, it is stated that $x$ $\epsilon$-constraint dominates solution $y$ (denoted as $x{ \prec _\epsilon }y$) if one of the following conditions is satisfied (Takahama, 2006):

-
$\phi (x) \; < \; \epsilon \;  \&  \; \phi\; (y) \;< \; \epsilon \;and\;x \prec y$,
-
$\phi (x) = \phi (y) = \epsilon \;and\;x \prec y$,
-
$\phi (x) \;> \; \epsilon \; \& \;\phi (y) \; > \; \epsilon \;and\;\phi (x) \prec \phi (y)$,

where $\epsilon$ denotes relaxation factor and $\phi (x)$ denotes overall constraint violation of $x$, $x \prec y$ denotes that $x$ pareto dominates $y$. $x \prec y$ means that $x$ is not worse than $y$ on all objective functions, and $x$ is better than $y$ on at least one objective function.

### Environmental selection strategies

The conventional environmental selection strategy employed in CMOCSO relies on the Euclidean distance archive truncation method. While the Euclidean distance metric offers certain benefits in quantifying spatial relationships among individuals, it fails to account for the distribution characteristics of the solution space. In particular, the distribution of individuals within the solution space may be uneven, a nuance that the euclidean distance does not adequately address. In contrast, the shifted density estimation (SDE) strategy (as referenced in Li, Yang & Liu, 2013) provides a more comprehensive evaluation of solution quality by considering both convergence and diversity. This approach has gained considerable traction in various multi-objective evolutionary algorithms (MOEAs) (as noted in Bai & Zhang (2024), Ou et al. (2024)). The fundamental premise of the SDE strategy is to assess the quality of an individual within the population based on the density of neighboring individuals within a specified spatial range. Specifically, the SDE strategy emphasizes the density surrounding an individual; a high surrounding density suggests that the solution represented by that individual is situated in a relatively crowded region, potentially indicating its superiority. Conversely, a low surrounding density may imply that the solution is located in a relatively sparse area, signaling a need for further exploration or enhancement.

The environmental selection process of the ICMOCSO algorithm follows these steps: First, calculate the fitness values of all individuals. Individuals with fitness values less than 1 are selected as the next generation population. If the number of the next generation population is equal to the preset population size, the environmental selection process ends. If the next generation population size is less than the preset population size, individuals with smaller fitness values are selected from unselected individuals to join the next generation population. If the next-generation population size exceeds the preset population size, individuals with higher shifted density are deleted until the next generation population size reaches the preset size.

### Complexity analysis

As previously discussed, the proposed ICMOCSO framework encompasses several key components, including the central-point moving strategy, grouping strategy, $stage1$, $stage2$, and the environment selection strategy. The time complexity associated with the central-point moving strategy is primarily influenced by the number of fitness evaluations, center-point calculations, and stage transition assessments, resulting in a complexity of $O(N)$. In contrast, the grouping strategy exhibits a time complexity of $O({N^2})$, which is largely determined by non-dominated sorting, as well as fitness value comparisons and calculations. The computational demands of the CMOCSO utilized in $stage1$ are characterized by a complexity of $O({N^3})$, while the main_Pop update employed in $stage2$ incurs a complexity of $O(m{N^2})$, as detailed in the original literature. Regarding the environment selection strategy, its fitness calculations and truncation operations are analogous to those found in SPEA2, thereby yielding a computational complexity of $O({N^2})$. In summary, the overall computational complexity of the ICMOCSO framework is $O({N^3})$.

### Proof of algorithm effectiveness

The experiments were conducted on a PC with Windows 10 operating system, an Intel(R) Core(TM) i5-9500 CPU @ 3.00 GHz, and 8 GB RAM, using MATLAB 2021b. The multi-objective optimization algorithm testing platform, PlatEMO (Tian et al., 2017) (version 4.2), was employed for performance comparisons.

To evaluate the performance of the ICMOCSO algorithm, we compared it with several other algorithms: CTSEA (Ming et al., 2021), CMOCSO (Ming et al., 2022a), MaOEAIT (Sun et al., 2019), RVEAa (Cheng et al., 2016), TriP (Ming et al., 2022b), and TiGE2 (Zhou et al., 2018). Each algorithm was run 30 times independently on 16 ZXH_CF (Zhou, Xiang & He, 2020) test sets to ensure the stability and reliability of the results. For fair performance comparisons, we used the hyper volume (HV) metric (Riquelme, Von Lücken & Baran, 2015) as the evaluation standard. HV is widely used in the field of multi-objective optimization as it comprehensively reflects the distribution and uniformity of the solution set. In the conducted experiment, we established several public parameters: the maximum number of iterations was set to 10,000, the population size was 100, the number of targets was determined to be 7, and the number of decision variables was set at 12. Additionally, the proposed algorithm was configured with a minimum value for the two-stage switching center shift of 0.005, a rate of decrease for the $\epsilon$ value ( $cp$) was controlled at a value of 2, a maximal generation ( ${T_c}$) of 900, and values for $\alpha$ and $\tau$ set at 0.95 and 0.05, respectively. For other algorithms, the parameters are set according to the original article, and the settings of public parameters are consistent with those in this article.

The HV metric results for the ZXH_CF test functions are detailed in Table 2. In Table 2, “+” indicates that the algorithm performed excellently compared to others, while “−” indicates poor performance. If there is no significant statistical difference, it is marked as “=”. The bold text indicates that the algorithm performed the best compared to others. From the data in Table 2, we observe that ICMOCSO performed slightly less well on ZXH_CF4, ZXH_CF7, ZXH_CF8, ZXH_CF10, and ZXH_CF13. However, it demonstrated superior performance on the other 11 test functions. This finding confirms the feasibility of the improvements made to CMOCSO and lays a solid foundation for the subsequent design of multi-PID controller parameter optimization algorithms.

**Table 2:** HV indicator.

Problem	CTSEA	CMOCSO	MaOEAIT	RVEAa	TiGE2	TriP	ICMOCSO
ZXH_CF1	4.2264e−1 (9.42e−2) −	7.9806e−1 (4.69e−2) −	3.9651e−1 (1.00e−1) −	9.4733e−1 (1.06e−2) −	8.9456e−1 (3.03e−2) −	4.9753e−1 (9.28e−2) −	**9.5599e−1 (5.57e−3)**
ZXH_CF2	2.4646e−2 (2.72e−2) −	2.5977e−1 (1.58e−1) −	4.0081e−2 (4.61e−2) −	5.7180e−1 (1.57e−1) −	2.7835e−1 (1.59e−1) −	6.0165e−2 (6.66e−2) −	**6.4786e−1 (1.29e−1)**
ZXH_CF3	1.0129e−4 (1.14e−4) −	1.0082e−3 (5.12e−4) −	8.6757e−4 (9.92e−4) −	6.6879e−3 (9.30e−4) −	6.7270e−3 (1.30e−3) −	3.7152e−3 (9.44e−4) −	**1.0264e−2 (4.33e−4)**
ZXH_CF4	**1.5206e−4 (3.15e−4) =**	1.6579e−5 (6.57e−5) −	8.3336e−4 (7.65e−4) =	7.6926e−4 (1.81e−3) =	6.8499e−5 (3.01e−4) =	4.4549e−5 (1.26e−4) −	3.6928e−4 (9.68e−4)
ZXH_CF5	5.9334e−4 (3.33e−4) −	3.4394e−4 (2.24e−4) −	1.2275e−4 (1.65e−4) −	1.0979e−3 (7.31e−4) −	6.8179e−4 (3.76e−4) −	3.3811e−4 (2.69e−4) −	**1.5143e−3 (7.45e−4)**
ZXH_CF6	3.8362e−4 (1.10e−4) −	6.0979e−4 (1.11e−4) −	7.5426e−6 (1.26e−5) −	1.0736e−3 (1.99e−4) −	9.9917e−4 (2.79e−4) −	3.7099e−4 (1.12e−4) −	**1.5620e−3 (1.27e−4)**
ZXH_CF7	**9.9114e−3 (1.16e−2) +**	0.0000e+0 (0.00e+0) =	NaN (NaN)	4.0600e−3 (8.89e−3) =	2.3042e−2 (3.02e−2) +	4.3936e−3 (1.26e−2) =	7.0625e−5 (2.00e−4)
ZXH_CF8	8.9130e−3 (1.17e−2) −	2.3600e−2 (1.33e−2) −	0.0000e+0 (0.00e+0) =	1.5968e−2 (8.92e−3) −	**1.4519e−1 (8.59e−3) +**	1.2195e−3 (3.37e−3) −	4.9869e−2 (1.56e−2)
ZXH_CF9	4.0858e−4 (2.50e−4) −	8.2989e−4 (3.18e−4) −	5.2850e−4 (5.14e−4) −	1.6335e−3 (6.38e−4) −	2.7949e−3 (9.34e−4) −	1.0814e−3 (7.25e−4) −	**4.3022e−3 (4.66e−4)**
ZXH_CF10	**1.2283e−4 (3.16e−4) +**	8.4872e−6 (4.30e−5) =	1.0850e−4 (2.66e−4) =	2.2829e−4 (5.99e−4) =	1.6816e−4 (6.39e−4) =	8.1215e−5 (3.37e−4) =	2.9210e−6 (1.35e−5)
ZXH_CF11	1.6800e−1 (7.17e−2) −	5.9411e−1 (3.25e−2) −	2.6048e−2 (2.83e−2) −	5.3872e−1 (4.58e−2) −	4.0467e−1 (8.63e−2) −	9.6920e−2 (5.15e−2) −	**6.3876e−1 (3.06e−2)**
ZXH_CF12	6.1705e−1 (7.83e−2) −	5.8588e−1 (2.99e−1) −	2.6009e−1 (1.01e−1) −	7.9503e−1 (1.11e−1) −	5.8342e−1 (1.89e−1) −	5.2983e−1 (1.28e−1) −	**9.1402e−1 (4.63e−2)**
ZXH_CF13	1.2018e−2 (2.17e−2) =	0.0000e+0 (0.00e+0) −	NaN (NaN)	**1.5175e−1 (1.82e−1) +**	3.0465e−2 (7.40e−2) +	2.4456e−3 (8.11e−3) =	4.6856e−3 (1.57e−2)
ZXH_CF14	4.3232e−1 (8.22e−2) −	8.4671e−1 (2.81e−2) −	2.3896e−1 (4.60e−2) −	9.2579e−1 (1.36e−2) −	8.7147e−1 (2.68e−2) −	4.6391e−1 (8.36e−2) −	**9.4096e−1 (8.05e−3)**
ZXH_CF15	4.5420e−4 (4.53e−4) −	8.5652e−4 (5.75e−4) −	4.7447e−4 (6.15e−4) −	2.5893e−3 (2.04e−3) −	1.1160e−3 (8.77e−4) −	2.3971e−3 (1.24e−3) −	**4.8744e−3 (2.28e−3)**
ZXH_CF16	3.0433e−4 (1.99e−4) −	2.4638e−3 (3.99e−4) −	1.2900e−4 (1.94e−4) −	8.1504e−3 (9.01e−4) −	9.6798e−3 (7.58e−4) −	2.4648e−3 (7.65e−4) −	**1.0639e−2 (5.27e−4)**
+/−/=	2/12/2	0/14/2	0/11/3	1/12/3	3/11/2	0/13/3	

**Note:**

The bold text indicates that the algorithm performed the best compared to others.

## Numerical simulation

Simulation experiments were conducted in the MATLAB 2021b/Simulink software environment. The algorithm described in this article was applied to the previously constructed simulation model of the hydraulic synchronization control system, and the results were thoroughly analyzed. To verify the superiority of the algorithm in optimizing multi-PID controller parameters, we compared it with several commonly used PID parameter optimization methods.

### Simulation setup

The parameter range for each individual in the constructed constrained multi-objective problem is 12-dimensional: ${K_{p1}},K_{p2},K_{p3},K_{p4}\in [22, 83.6]$, $K_{i1},K_{i2},K_{i3},K_{i4}\in [0,894]$, $K_{d1},K_{d2},K_{d3},K_{d4}\in [-0.8,0.8]$. Simulation experiment parameters: simulation time is $1s$, population size is 100, maximum iterations are 10,000, maximum overshoot ( ${\sigma _{max}}$) is 1, lower bound of gain margin ( ${p_{min}}$) is 6, the upper bound of phase margin ( ${g_{max}}$) is 60, and the lower bound of phase margin $({p_{max}}$) is 30.

### Result analysis

The ICMOCSO algorithm can obtain the optimal solution set with a single run. From the optimal solution set, we selected three representative solutions for analysis, as shown in Table 3. In Table 3. the first solution set focuses on minimizing synchronization error, the second set on minimizing overshoot, and the third set on minimizing rise time. The bold indicates the best performance in the respective criterion compared to other solutions.

**Table 3:** Part of representative solutions and their performances.

	1	2	3
master_PID_1	[22, 0, 0.019265]	[22, 0, 0.095881]	[80.063, 15.533, −0.47181]
slave_PID_2	[82.267, 0, −0.45424]	[83.027, 0, −0.57822]	[68.913, 108.14, −0.53624]
slave_PID_3	[83.6, 585.28, −0.44728]	[83.6, 702.49, −0.63137]	[55.900, 52.561, −0.25372]
slave_PID_4	[57.714, 330.49, −0.13852]	[58.602, 260.62, −0.43035]	[82.522, 0, −0.65188]
${e_{ss}}$	0.000421	0.000449	0.000916
$\sigma$	0.054185	0.034680	1.505584
*t_r_*	0.256920	0.280591	0.101328
*ITSE*	0.000380	0.000403	0.001064
${t_s}$	0.553277	0.543328	0.381143
${t_m}$	1.648110	1.170193	0.133254
$se$	0.000003	0.000007	0.000138

According to Table 3, we can observe that the three sets of solutions exhibit different characteristics in terms of performance. Specifically, the first solution set performs best in terms of synchronization error and has a better steady-state error compared to the other two sets, though other performance metrics are inferior. The second set of solutions has the smallest overshoot, but its other performance metrics are relatively poor. The third solution set has the shortest rise time, and except for steady-state error, its other performance metrics are superior to the other two sets. This finding reveals that in the process of constrained multi-objective optimization of multi-PID controller parameters, we can achieve diverse control effects to meet various control requirements. For different control requirements, we can flexibly select and adjust the parameters of the multi-PID controller to achieve the optimal control effect.

### Comparison with other methods

The model constructed in this article is a constrained multi-objective model. Compared with other multi-objective algorithms, constraints are transformed into two objective functions, ${f_{less}}$ and ${f_{greater}}$. For any individual $i$ in the population, the constraints are handled using the following method.

(16) $${f_{greater}}(i) = {{GM(i) - {g_{\min }}} \over {\max (GM(i) - {g_{\min }})}} + {{PM(i) - {p_{\min }}} \over {\max (PM(i) - {p_{\min }})}}$$

(17) $${f_{less}}(i) = - {{PM(i) - {p_{\max }}} \over {\max (PM(i) - {p_{\max }})}} + {{{\sigma ^c}(i) - {\sigma _{\max }}} \over {\max ({\sigma ^c}(i) - {\sigma _{\max }})}}$$

In Eqs. (14) and (15), *GM* represents the gain margin, *PM* represents the phase margin, ${g_{min}},{p_{max}},{p_{min}}$ are their respective bounds, ${\sigma ^c}$ denotes the overshoot, and ${\sigma _{max}}$ is the upper limit of the overshoot.

After processing the constraints, the constructed constrained multi-objective model is transformed into a multi-objective optimization model with nine objectives. Therefore, we chose the upgraded NSGA-III algorithm, suitable for many-objective optimization, as a comparison algorithm.

The method proposed in this article is compared with other PID parameter optimization methods, including the Ziegler-Nichols (Z-N) (Ziegler & Nichols, 1942), the PID parameter tuning method using MATLAB’s built-in Rltool toolbox (Zhang et al., 2016), the NSGA-III method (Deb & Jain, 2013), the GFMMOEA method (Tian et al., 2018), the MaOEAIT method (Sun et al., 2019), and the CMOCSO method (Ming et al., 2022a). In the traditional PID method and the Rltool toolbox method, the master and slave cylinder controllers use the same PID parameters. Among the optimal PID parameter solutions obtained by other methods, to ensure a fair and comprehensive comparison, we selected a solution that performs relatively well in various performance metrics (*i.e*., a knee point Zhang, Tian & Jin, 2014) for comparative experiments.

The detailed PID controller parameters and their performance are shown in Table 4. In the Table 4, “+” indicates that the optimization method is superior to the other comparison methods in a particular performance metric, while “−” indicates that the method is inferior.

**Table 4:** Parameters and performance of PID controllers with different optimization methods.

	Z-N	Rltool	NSGAIII	GFMMOEA	CMOCSO	MaOEAIT	ICMOCSO
master_PID_1	[20, 1, 0.1]	[43.88, 595.62, −0.069858]	[58.701, 1.5912, −0.13832]	[62.243, 6.6812, −0.38809]	[44.584, 235.28, −0.030137]	[34.396, 809.64, 0.018892]	[30.1042, 0, 0.13175]
slave_PID_2	[20, 1, 0.1]	[43.88, 595.62, −0.069858]	[57.478, 0, −0.4663]	[39.008, 19.988, −0.25582]	[83.534, 385.69, −0.60278]	[64.682, 423.37, −0.2]	[80.6895, 31.7712, −0.6360]
slave_PID_3	[20, 1, 0.1]	[43.88, 595.62, −0.069858]	[62.761, 1.137, −0.52684]	[41.464, 15.157,−0.14808]	[78.556, 564.77, −0.66593]	[71.1, 233.7, −0.4]	[83.2205, 659.5338, −0.5744]
slave_PID_4	[20, 1, 0.1]	[43.88, 595.62, −0.069858]	[56.718, 29.33, −0.44164]	[31.018, 0, −0.091883]	[74.424, 286.51, −0.5771]	[69.1, 520.1, −0.3]	[54.4354, 155.89, −0.49727]
${e_{ss}}$	0.001080 −	0.000908 −	0.000673 −	0.000814 −	0.000734 −	0.000735 −	0.000618
$\sigma$	0.000391 +	1.221087 −	0.691222 −	0.372254 −	0.916052 −	1.372528 −	0.096760
$t_r$	0.472331 −	0.114674 +	0.111628 +	0.149212 −	0.104596 +	0.105947 +	0.147060
*ITSE*	0.001357 −	0.000988 −	0.000719 −	0.000941 −	0.000643 −	0.000709 −	0.000478
${t_s}$	0.725853 −	0.801046 −	0.383907 +	0.263743 +	1.305834 −	0.620071 −	0.5204941
${t_m}$	2.248877 −	0.208799 +	0.205223 +	0.233068 +	0.169461 +	0.220007 +	0.314852
${se}$	0.0001183 −	0.0000003 +	0.0000537 −	0.000144 +	0.0000250 +	0.000013 +	0.0000464

From the table data, we can observe that the optimization method proposed in this article improves performance metrics across the board compared to the traditional PID method, except for overshoot. Compared to the Rltool method, the ICMOCSO method demonstrates better performance in synchronization error, overshoot, settlement time, and integral time square error. Compared to the NSGA-III method, the ICMOCSO method shows advantages in synchronization error, overshoot, steady-state error, and integral time square error. Against the GFMMOEA method, the ICMOCSO method excels in synchronization error, overshoot, rise time, and integral time square error. Compared to the CMOCSO method, the ICMOCSO method outperforms in synchronization error, overshoot, settling time, and integral time square error. Finally, compared to the MaOEAIT method, the ICMOCSO method shows better results in synchronization error, overshoot, settling time, and integral time square error. In summary, the comparative experiments indicate that, on average, the ICMOCSO method reduces the synchronization error by approximately 23.28% and the integral time square error by approximately 46.21% compared to other PID parameter optimization methods.

**Figure 5:**
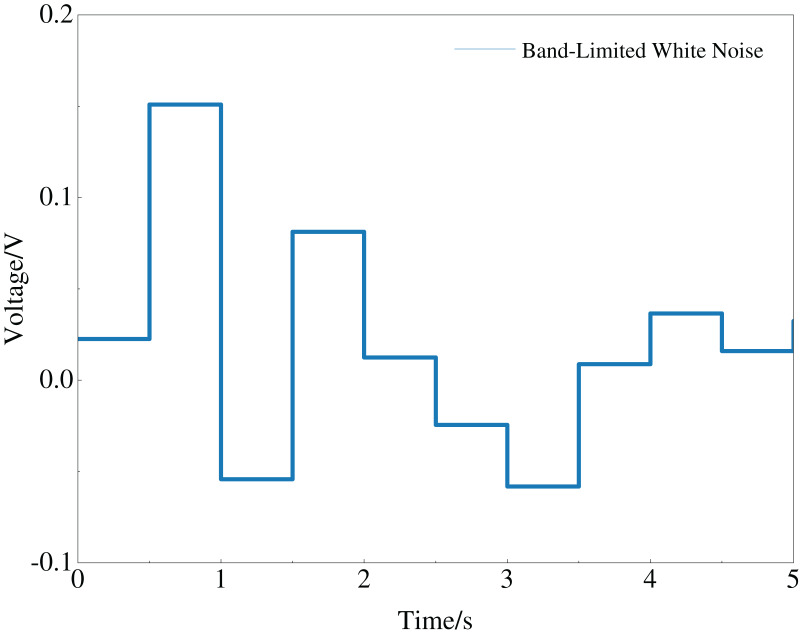
General principle of improved CMOCSO.

When constructing the constrained multi-objective problem model for the multi-PID controller, frequency domain indicators were considered to ensure that the optimized PID controller parameters possess anti-interference capability. To test this anti-interference capability, white noise with a noise power of 0.002 and a sampling time of 0.1s was added to the system. The white noise is shown in Fig. 5.

**Figure 6:**
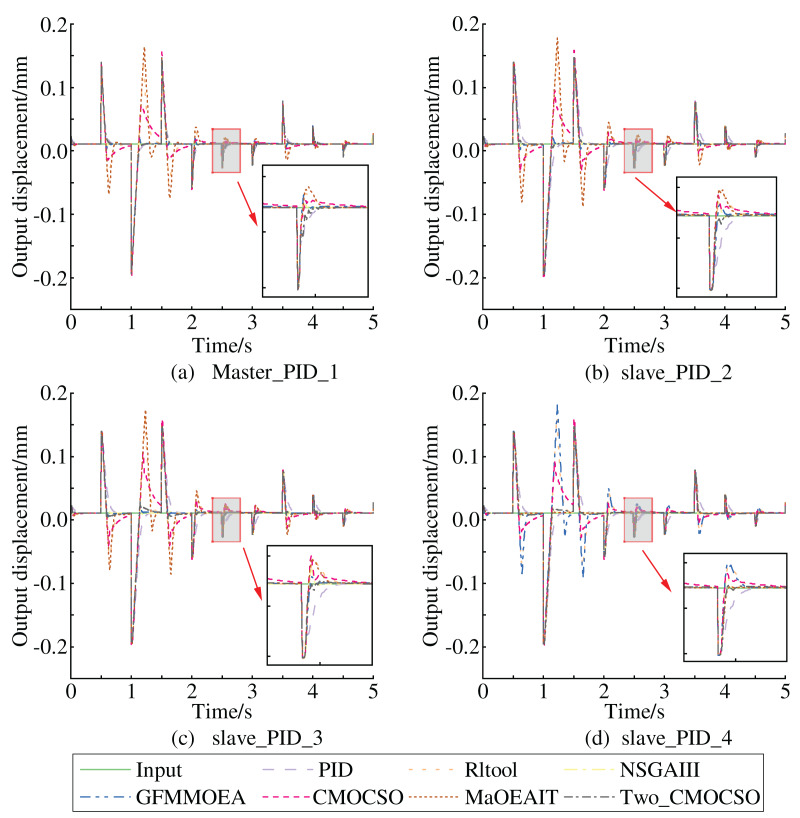
General principle of improved CMOCSO.

Incorporating the white noise into the simulation model, we again observed the step response curves of each subsystem, as shown in Fig. 6. In Fig. 6, it is clear that under noise interference with a power of 0.002, the multi-PID controller subsystems optimized by the ICMOCSO method, except for the main structure’s PID controller subsystem, reached and maintained within the final steady-state value’s 2% error band the fastest. This demonstrates that the PID parameters optimized by the ICMOCSO method have excellent anti-interference capability, significantly reducing the impact of external disturbances on the system.

## Conclusions

To minimize the synchronization error and enhance the anti-interference capability of the multi-PID controller system, a solution is proposed in this article: a multi-PID controller parameter optimization approach based on the ICMOCSO algorithm. Initially, the parameter optimization issue of the multi-PID controller is transformed into a constrained multi-objective optimization problem. Subsequently, the ICMOCSO algorithm is presented. This algorithm, through the center-point movement strategy, divides the optimization process into two phases, each of which adopts targeted evolution strategies to accelerate convergence and improve the quality of the solution. Moreover, a grouping strategy is introduced to further strengthen the global exploration and local exploitation capabilities of the algorithm. The experimental results on 16 standard test functions such as ZXH_CF reveal that the ICMOCSO algorithm demonstrates outstanding diversity and convergence in addressing the seven-objective constrained multi-objective optimization problem, validating its effectiveness and superiority.

More significantly, the ICMOCSO algorithm is applied to the optimization practice of multi-PID controller parameters. Simulation experiments indicate that through a single run, this algorithm can generate a set comprising multiple potential optimal solutions, offering flexible options for different control requirements and achieving the adjustment of the optimal control effect. Compared with other methods, the average value of the synchronization error of the ICMOCSO method decreases by approximately 23.28%, and the average value of the integral time square error drops by about 46.21%, while showing good anti-interference ability. These advantages endow the ICMOCSO algorithm with a higher application value and stronger robustness in actual industrial control systems.

Despite the outstanding performance of the ICMOCSO algorithm in multiple aspects, it also encounters certain limitations and challenges. Firstly, when dealing with large-scale or high-dimensional problems, the algorithm might require more computing resources and time. Furthermore, although the algorithm performs well in the parameter optimization of PID controllers, its application might be limited to specific control systems and optimization problems. For other types of issues, further adjustments or validations might be necessary. In response to the above challenges, future research will focus on enhancing the universality and efficiency of the algorithm, verifying its performance in various actual industrial control systems, and exploring effective combination strategies with other advanced optimization algorithms.

## Supplemental Information

10.7717/peerj-cs.2453/supp-1 Supplemental Information 1 NSGAIII: Experimental results of NSGAIII algorithm under multi-PID controller model.

10.7717/peerj-cs.2453/supp-2 Supplemental Information 2 Two CMOCSO Algorithm.

10.7717/peerj-cs.2453/supp-3 Supplemental Information 3 GFMMOEA: Experimental results of GFMMOEA algorithm under multi-PID controller model.

10.7717/peerj-cs.2453/supp-4 Supplemental Information 4 CMOCSO: Experimental results of CMOCSO algorithm under multi-PID controller model.

10.7717/peerj-cs.2453/supp-5 Supplemental Information 5 MaOEAIT: Experimental results of MaOEAIT algorithm under multi-PID controller model.

10.7717/peerj-cs.2453/supp-6 Supplemental Information 6 Two CMOCSO element: Experimental results of Two_CMOCSO algorithm under multi-PID controller model.

10.7717/peerj-cs.2453/supp-7 Supplemental Information 7 Experimental results of the algorithm on NXH-CF test function.

10.7717/peerj-cs.2453/supp-8 Supplemental Information 8 MODEL: Multi-PID controller mathematical model code.

10.7717/peerj-cs.2453/supp-9 Supplemental Information 9 ZXH CF test function code.

## Additional Information and Declarations

### Data Availability

The following information was supplied regarding data availability: The source code is available in the Supplemental File.
